# Sinus Membrane Elevation with Heterologous Cortical Lamina: A Randomized Study of a New Surgical Technique for Maxillary Sinus Floor Augmentation without Bone Graft

**DOI:** 10.3390/ma11081457

**Published:** 2018-08-17

**Authors:** Antonio Scarano, Pablo Santos de Oliveira, Tonino Traini, Felice Lorusso

**Affiliations:** 1 Department of Medical, Oral and Biotechnological Sciences and CeSi-Met, ‘G. D’Annunzio’ University of Chieti-Pescara, Via dei Vestini 31, 66100 Chieti, Italy; t.traini@unich.it; 2 Department of Oral Implantology, Dental Research Division, College Ingà, UNINGÁ, Cachoeiro de Itapemirim, 29312 Espirito Santo, Brazil; psoliveiraodonto@yahoo.com.br; 3 Department of Medical, Oral and Biotechnological Sciences University of Chieti-Pescara, Via dei Vestini 31, 66100 Chieti, Italy; drlorussofelice@gmail.com

**Keywords:** histomorphometry, cone beam computed tomography, collagenated porcine bone, resorbable barrier, sinus lift, heterologous cortical lamina, without graft

## Abstract

Background: The aim of this randomized controlled clinical trial was to compare the efficacy of two different techniques for maxillary sinus augmentation using a lateral window approach: Heterologous cortical lamina without any grafting material versus 100% collagenated granular collagen porcine bone. Methods: Twenty-three healthy patients with not relevant past medical history (14 women and 9 men, non-smokers, mean age 52 years, range 48–65 years) were included. In Group I, the sinus was filled with collagen porcine bone (Geno-os, OsteoBiol, Turin, Italy) and a collagen membrane (Evolution, OsteoBiol, Turin, Italy) was used to close the lateral window of the sinus. In Group II, the sinus was treated with heterologous cortical lamina only (Lamina, OsteoBiol, Turin, Italy). Results: There was a statistically significant difference in the surgical time required to complete the augmentation procedures: 18.3 ± 2.1 min for lamina treated sites versus 12.5 ± 3.1 min for porcine bone treated sites. In Group I, the mean volume of the graft was 3101 ± 321 mm^3^ in the immediate postoperative examination (5–7 days), while after a six-month healing period it was 2716.7 ± 432 mm^3^. Conclusion: This study demonstrates that the use of heterologous cortical lamina is a valid technique for the mechanical support of sinus membranes resulting in only bone tissue formation and not mixed with the graft. The graft material was biocompatible and not completely resorbed after six months, although the remains were integrated into the bone.

## 1. Introduction

Implant placements in areas of deficient ridge width or depth often result in exposure of the fixture threads and formation of implant-associated defects. The treatment of edentulous posterior maxilla with implant supported rehabilitation may result difficult for the insufficient bone volume in consequence of buccolingual and/or apical occlusal atrophy of the edentulous alveolar crestal bone and pneumatization of the maxillary sinus. In this anatomical condition, the implant primary stability can be very difficult to obtain in absence of a useful quantity of cortical bone and for the loose structure of type IV spongious bone which prevents the fixture migration into the sinus [1,2]. Many different surgical techniques have been developed to treat the atrophic posterior maxilla. For some decades, sinus augmentation procedures performed using several bone grafts has been proposed to treat the posterior maxilla for dental implant placement [3,4]. The use of allografts, xenografts and alloplasts has been reported in the literature and they help bone formation. Most of these biomaterials have osteoconductive properties and represent scaffolds that guide bone formation [5]. Their availability is a useful advantage of these materials. Xenografts have been reported to show a similarity to human bone tissue morphology [6].

Bone substitutes should ideally possess osteoinductive, osteoconductive, and osteogenic properties. To perform the formation of vital bone, implants need to integrate in newly-formed bone and have long-term survival after functional load. These properties vary in different bone grafting materials [7,8,9]. Autogenous bone is considered ideal as an augmentation material. Previous studies were performed regarding the benefits of bone xenografts for the augmentation of maxilla sinus, with positive results. Grafting materials that are utilized in floor augmentation could provide for a bone formation process, by replacing the bone materials, due to capillary infiltration, and support of the implants [10]. These grafting materials help to maintain space between the basal bone and membrane.

When augmentation is achieved after surgical procedure, the possibility of new bone formation with a membrane elevation in the maxillary sinus has been reported in human and animal studies [11,12]. The aim of this randomized controlled clinical trial was to compare the volumetric and histomorphometric properties of two techniques for maxillary sinus augmentation using a lateral window approach: heterologous cortical lamina without any grafting material versus 100% collagenated granular porcine bone. The present investigation is a preliminary report focusing on outcomes up to six months after healing.

## 2. Results

### 2.1. Group I

**Figure 1:**
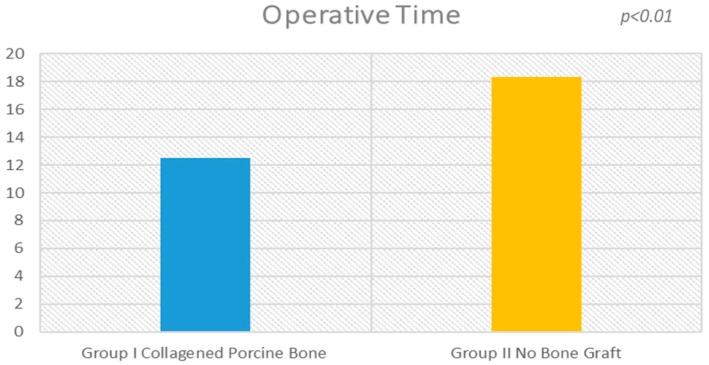
There is a statistical difference (*p* < 0.01) between the operative times of the procedure between the experimental groups.

**Figure 2:**
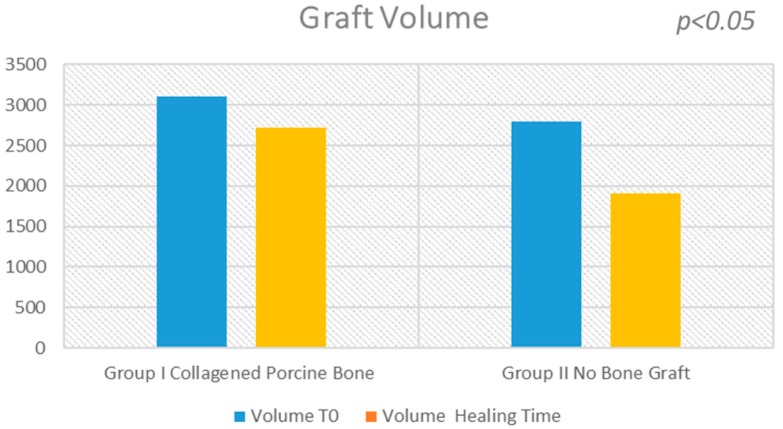
The evaluation shows a high statistical difference between the Group I (collagened porcine bone) and the Group II (no bone graft). At six months, the sinus bone gain in the non-graft group was significantly lower than in the graft group (504.6 mm^3^; 95% CI: 406.5 to 602.6; *p* < 0.05).

**Figure 3:**
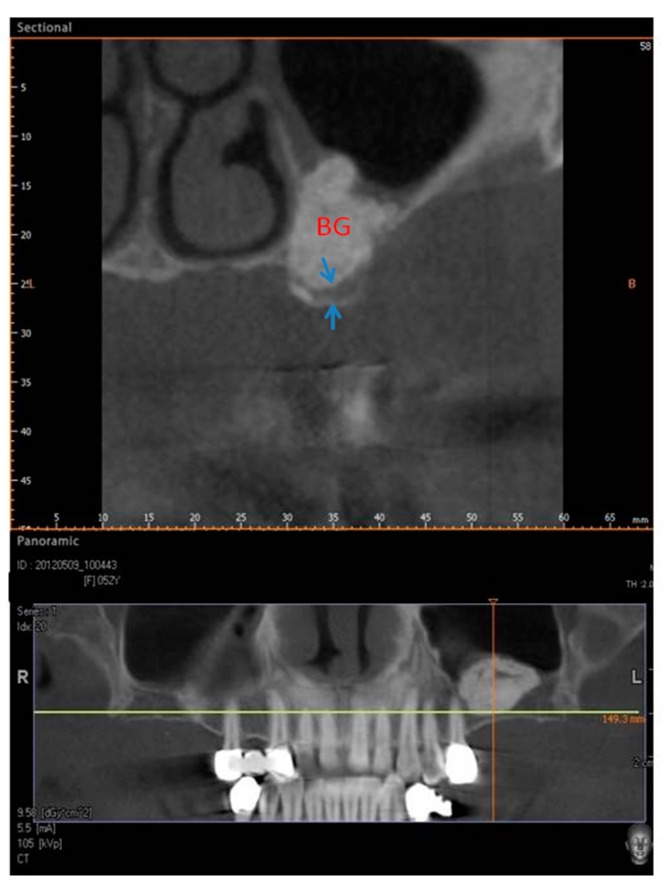
Group I. Six months after sinus lifting. CBCT image showing reduced bone height in the sinus area with a residual alveolar ridge height between 1 and 3 mm (arrows) and bone graft (BG).

The surgical time required to complete the augmentation procedures was 12.5 ± 3.1 min calculated for a total of 14 sinuses (Figure 1). The mean volume graft was 3101 ± 321 mm^3^ immediately postoperative and 2716.7 ± 432 mm^3^ after six months of healing (Figure 2). Forty-five Cone Beam Computer Tomography (CBCT) scans of the sinus augmentation were evaluated and no perforation of the sinus membrane was present in the 13 sites treated, while in 1 sinus a small membrane perforation was evident. Clinically, no fever, pain or acute infection was observed in all cases treated. Radiographically, bone grafts showed increased hyperdensity in comparison between immediate postoperative and after six months healing, with higher density than native bone (Figure 3). In the second surgical phase, the sinus wall was found to be totally healed in all cases. During bone core biopsies retrieved with a trephine, the healing of the sinus was obvious. No gaps were present at the bone–porcine bone interface that was always in close contact with the graft particles (Figure 3). Porcine bone granules presented marked staining differences from the host bone and had a decreased affinity for the stains.

**Figure 4:**
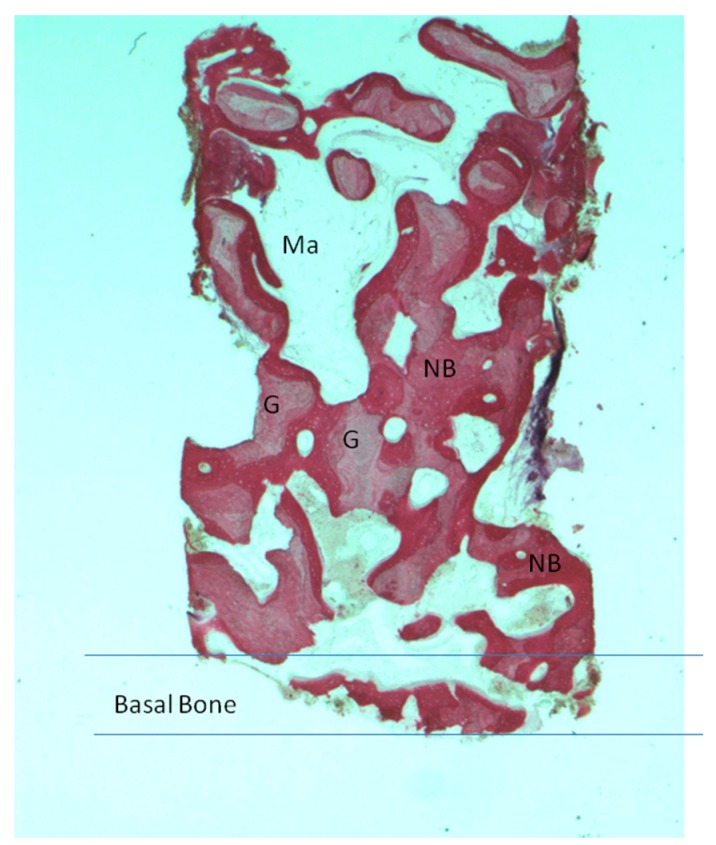
Group I. New bone extended from the basal bone of sinus. The graft (G) was surrounded by new bone (NB). A large madullary space was present (Ma). Acid fuchsin and toluidine blue 4X (X represents the power magnification of microscopical image).

Newly formed bone with wide osteocyte lacunae and large marrow spaces were present with newly formed vessels and no inflammatory cell infiltrate (Figure 4). In a few areas, it was possible to see a rim of osteoblasts, while no osteoclasts or macrophages cells were present. No resorption evidence was present in the sample. In a few areas, it was possible to observe the presence of compact, mature cortical bone, which could be easily differentiated from the newly formed bone. The tissues present in the sample were composed of 34 ± 1% new bone, 38 ± 3% marrow space, 35 ± 4% residual biomaterials and 3.02 ± 2% osteoid tissue.

### 2.2. Group II

**Figure 5:**
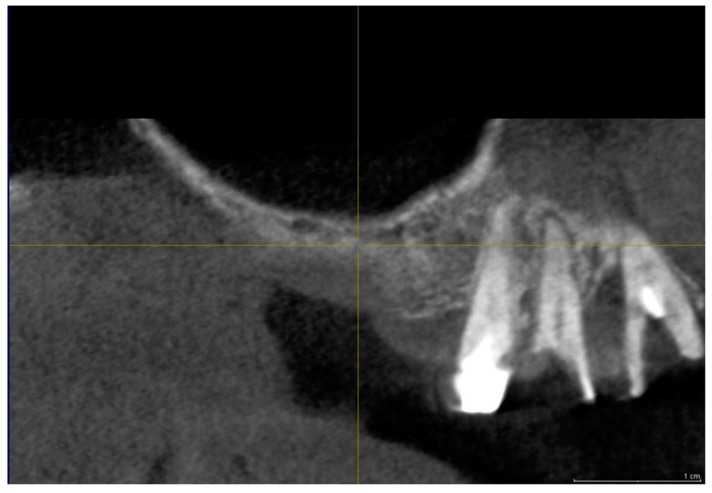
Group II. CBCT image before sinus lifting showing reduced bone height in the sinus area with a residual alveolar ridge height between 1 and 3 mm.

**Figure 6:**
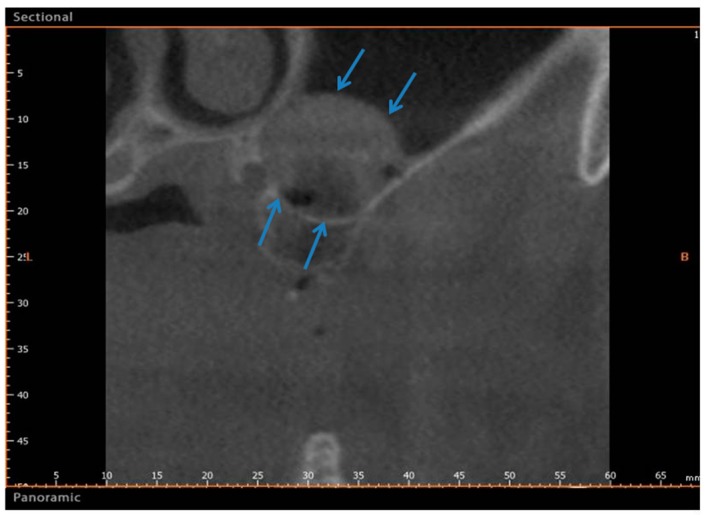
Group II. After six months, CBCT image showing a bone formation in the sinus (Arrows). The arrows indicate the newly formed bone between the basal bone and the upper portion of sinus.

The surgical time required to complete the augmentation procedures was 18.3 ± 2.1 min for a total of 14 sinus augmentations, Figure 1, Figure 5 and Figure 6. The mean volume graft was 2801 ± 215 mm^3^ immediate postoperative and after six months of healing it was 1912.1 ± 332 mm^3^ (Figure 2). Forty-five CBCT scans of the treated sinus were performed at different times. Clinically, no perforation of the sinus membrane was evident in 11 sites, while in 2 sites a small perforation was present in correspondence to the site of the surgery. In particular, one rupture of the lining was induced by incorrect handling of the heterologous cortical lamina membrane. The Schneiderian membrane was perforated during positioning of the cortical lamina by a cutting edge not correctly reduced by the operator. The perforation at the porcine bone site was sealed with a cortical lamina. In all cases, no acute infection, pain or fever was observed after treatment.

**Figure 7:**
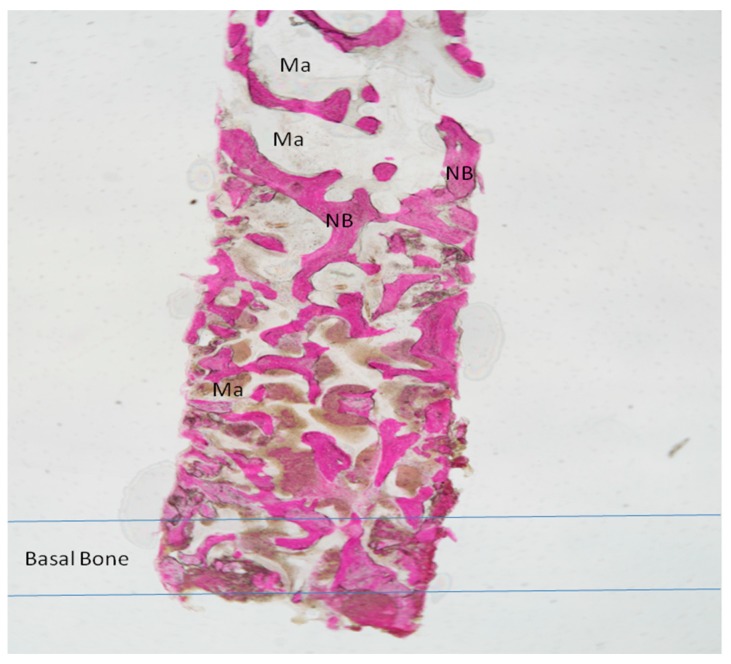
Group II. New Bone trabeculae (NB) were seen throughout the large marrow spaces (Ma). The basal bone with a small marrow spaces. Acid fuchsin and toluidine blue 4X.

In all subjects, bone augmentation tomographical analysis showed increased hyperdensity in comparison between the immediate postoperative examination and after six months of healing, with increased density of native bone (Figure 6). In the cortical lamina group, newly formed bone with wide osteocyte lacunae and large marrow spaces were present histologically with newly formed vessels (Figure 7). No pathological inflammatory cell infiltrates or foreign body reactions were evident and few osteoblasts were present in the specimens evaluated. At low magnification, the samples showed trabecular bone without epithelial cells or connective tissue, while prominent woven and mature bone were found. Mature bone originating from the endosteal surface filled the external portion of the bone sinus (Figure 7). The periphery and central portion of the cavities showed mineralized tissues and new bone formation. In all subject, the sites appeared completely healed in second surgical phase. Osteoid matrix produced by osteoblasts activity were observed and a moderate quantity of marrow stromal cells and vascular networks contained in marrow spaces. In particular, seams of osteoblasts and unmineralized matrix with collagen fibrils were observed in close proximity to new bone apposition areas. The tissues present in the sample were composed of 27 ± 4% new bone, 61 ± 3% of marrow space and 2.68 ± 3% osteoid tissue.

### 2.3. Statistical Analysis

There was statistically significant difference in the surgical time required to complete the augmentation procedures: 18.3 ± 2.1 min for lamina treated sites versus 12.5 ± 3.1 min for porcine bone treated sites (Figure 1 and Figure 2), which is significantly greater in the non-grafted sinus one (5.1; 95% CI: 3.7430–7.8570; *p* < 0.01).

The statistical analysis showed a significant difference of volume change before and after sinus lifting (*p* = 0.001229) for each group. A small statistical difference was found in the volume change between sinus lifting and after six months healing in Group I (−384.300 mm^3^, 95% CI; −679.9703 to −88.6297; *p* = 0.0165). A large statistical difference was found in the volume change between sinus lifting and after six months of healing in Group II (−888.900 mm^3^, 95% CI; −1106.1932 to −671.6068; *p* < 0.01).

The histological results showed new bone percentages in both groups. A statistically significant difference was evident in the percentages of bone formation (7.0%, 95% CI; 8.2656 to 5.7344, *p* = 0.006) and a large difference was present in the percentages of marrow space (31.320%, 95% CI; −32.8548 to −29.7852, *p* = 0.000001). The histomorphometric results showed that, at the observed time, the amount of marrow spaces and residual graft material more than new bone formation are always quite different from 100%, which is because the three measurements were carried out individually with a margin of error, thus the sum of the error includes measurements in the graft, bone, and woven bone and marrow spaces.

## 3. Discussion

The outcomes of the present study show that sinus membrane elevation, with or without bone graft, presents good results in a six-month follow-up, but in the sinus treated with bone lamina there was greater volumetric contraction. In addition, the surgical time required to complete the augmentation procedures was major compared to the traditional technique. This study also demonstrates that the use of heterologous cortical lamina is a valid technique for mechanical support of the sinus membrane resulting in only bone tissue formation and not mixed with graft. The graft material was biocompatible but not completely resorbed after six months, although the remains were integrated into the bone.

This study demonstrates a low incidence of perforation of sinus membrane. Complications occurred in two patients of the heterologous membrane group versus one complication in the porcine bone group. All complications occurred during the sinus augmentation procedure and consisted of rupture of the sinus membrane. These results agree with previous studies [3,13] that have shown that the simple elevation of the Schneiderian membrane can induce bone formation in the maxillary sinus. However, the results of this work provide more information on the volumetric contraction in sinus lifting without bone graft during a six-month healing period.

Blood supply and angiogenesis hold a key role in new bone formation. Indeed, a blood clot contains a great quantity of growth factors (GFs) in its naturally-occurring and biologically determined ratio and are successful in acute wound healing. These GFs include: vascular endothelial growth factor (VEGF), platelet-derived growth factor (PDGF), epidermal growth factor (EGF), insulin-like growth factor-1 (IGF-1), basic fibroblast growth factor (bFGF), transforming growth factor-b1 (TGF-b1), and transforming growth factor a (TGF-a) [14,15]. These molecules have the ability to interact with cells such as osteoblasts endothelial cells and stem cells in the subcutaneous tissues. They can activate intracellular signaling events mediating cell proliferation, migration, survival and production of extracellular matrix proteins after binding to their cellular receptors [16,17].

Probably, the mechanisms subtending the bone formation that play a key role are a migration of osteopotent cells from the denuded bone walls and osteotomy access in response to surgical trauma. Spontaneous sinus bone formation has been reported after removal of a migrated dental implant [18] or cyst [19] and an extraction socket [20]. These studies observed a great potential for healing and new bone apposition in the maxillary sinus without the use of additional grafts and substitutes. This outcome was related to the concept that the lifting of the Schneiderian membrane and the establishment of a compartment with a blood clot could induce new bone formation around the inserted implants in a similar way to which bone biomaterials do to maintain the augmented space and promote osteogenesis [21].

Another hypothesis is that the sinus mucosa periosteum contributed to the reaction and new bone apposition. These osteogenic properties have been recently observed through a study in vivo and in vitro [22]. The key factor of new bone apposition is the osteogenic capability of the sinus mucosa that includes periosteum [23]. Surgical trauma and the formation of a regenerative compartment between the bone walls and the Schneiderian membrane result in a spontaneous bone apposition in the maxillary sinus. However different studies have shown that bone is forming from the denudated bone walls of the sinus while the sinus mucosa did not show evident signs of bone formation [24,25,26,27]. In fact, in the present study, the healing of the augmented sinus was also achieved in the group that used cortical lamina so that the partial exclusion of the sinus mucosa from the healing bone process seems not to have affected the results. This outcome is in agreement with the results from other reports on the healing in augmented sinus in sheep and rabbits in which the sinus mucosa was excluded from the elevated space by the use of collagen membranes [28,29,30].

The sinus membrane elevation procedure was investigated in patients referred for sinus augmentation [6,10].

Maxillary sinus augmentation and bone regenerative protocols present similarities because they are coordinated processes involving many biological factors [15,31]. The outcome of our study showed that the maxillary sinus presents a great potential for bone healing beyond the skeletal contour, as a response to a surgical trauma. In part, this evidence may clarify the good results with sinus floor augmentation protocols and suggests that the role of bone biomaterials and substitutes to realize new bone formation in the maxillary sinus may have been overemphasized [6,10,32].

Sinus membrane stabilization is a requirement for bone formation. It must be pointed out that sinus pneumatization could represent a consequence of positive intrasinus air pressure produced by respiration, and this pressure might induce bone resorption and new pneumatization after sinus augmentation procedure [33,34]. To avoid this, Borges et al. [13] pushed the lateral bone window inside the sinus cavity, using this thin bone as the “roof” of the secluded cavity. The authors used implants for a mechanically supported bone window to preserve a space maker for guided bone regeneration [35]. The limitation of this technique is the distance between the antrostomy and the nose wall; in fact, in the case of a large distance, the bone window is insufficient for supporting the sinus membranes. Many anatomical factors are able to influence new bone formation; in fact, the bone formation processes after sinus lifting are negatively correlated with reduced crestal height [24,36], the width of the bucco-palatal sinus [37] and the width of the bony window during lateral sinus augmentation [24,36].

In the present study, we used a cortical bone lamina of 1 mm thickness, modeled and positioned in the sinus as a new roof sinus. This membrane is rigid and stability improves through the two lines of 2–3 mm, mesial and distal, created at the top of the antrostomy. The advantage of this technique is that it obtains only bone, without residual biomaterials. In fact, the outcome of this research shows a bone formation with a large marrow space. Previous studies have demonstrated that all tested materials can be used as grafts in maxillary sinus augmentation procedures and appear to be well tolerated in all cases and highly biocompatible.

At the experimental times, the histomorphometric outcome showed that differences between the materials related to the quantity of marrow spaces and residual graft were more than to new bone formation [38]. Different complications, including the failure of the graft, may occur after sinus augmentation, e.g. infection, displacement of the implant inside the maxillary sinus, insufficient bone quantity apposition to allow implant insertion, and the formation of an oroantral fistula [39]. In the case of infection, the grafted augmented sinuses should be treated early and aggressively to avoid bacterial proliferation to biomaterials. An infection of the graft biomaterials can also happen in occurrence of peri-implantitis upon dental implants on grafted sinuses [40]. The results also suggest that these benefits may be at the cost of increased operating time of sinus augmentation without graft. Today, the majority of clinical surgeons use bone grafts for filling sinuses.

The disadvantage of this technique is represented by the isolation of the sinus membrane which cancels its osteogenetic capacity, a greater volumetric contraction and a longer time required to complete the augmentation procedures when compared to the traditional technique. Indeed, it has been shown that cells deriving from explants of sinus membranes can express markers of osteoprogenitor cells [22]. This suggests native, latent osteogenic activity of the Schneiderian membrane, but the cellular basis for this presumed activity is unclear. The osteogenesis process requests viable active osteoblasts (bone forming cells) derived from mesenchymal progenitors [41,42]. These progenitors are naturally present in the bone marrow stroma and periosteum, where they are extensively characterized, and in other tissues, such as in adipose one and microvascular walls [43]. The virtual paucity of biological studies of the presumed osteogenic potential associated with the sinus membrane has contributed to hide the debate about its potential significance in order of a clinical application with uncertainty. Gruber et al. observed that cells deriving from the porcine sinus associated mucosa express STRO-1, a marker of osteoprogenitor cells, and respond to BMP-6 and BMP-7 [44]. The technique proposed in this paper of mechanically supported sinus membrane for space making overcomes the anatomic limits of other techniques. In conclusion, the surgical trauma and the creation of an excluded compartment between the bone walls and the healed Schneiderian membrane resulted in a spontaneous bone formation in the maxillary sinus. In fact, the outcome of this study showed good bone formation in both groups and higher in the graft-treated group. This result could be understood if we evaluated only the greater percentage of newly formed bone in the group treated with graft, but in group without graft we have a greater percentage of bone marrow spaces where ostoprogenitor cells are present and we have no biomaterial residues.

In conclusion, the surgical approach described may be used to achieve bone formation to enable placement of dental implants without the addition of any grafting material.

## 4. Materials and Methods

The study was conducted in observance of the Helsinki Declaration (revised version of Tokyo in 2004) and Good Clinical Practice Guidelines. The trial was approved by the Inter Institutional Ethics Committee of Faculdade Ingá, UNINGÁ, PR, Brazil, N 89018318.2.0000.5220.

A total of twenty-three healthy subjects with non-contributory past medical history (14 women and 9 men, all non-smokers, mean age 52 years, range 48–65 years) were included in this study where a total 28 maxillary sinuses were treated. The candidates were selected for sinus augmentation in the posterior maxilla in order to receive implant supported rehabilitation, and signed a written informed consent. The surgery was performed in the Outpatient Department of Oral Implantology, Center for advanced studies, Dental Research Division, UNINGÁ-Cachoeiro de Itapemirim, Brazil. A total of 18 patients were treated for unilateral sinus augmentation, while in 5 patients the procedure was bilateral for a total 28 surgical sites. Moreover, the sites were randomly allotted into two different groups, with 14 sinuses each, and the procedures were performed by a single surgeon. The inclusion criteria provide for fully edentulous or partially edentulous subjects affected by unilateral or bilateral loss of teeth in the maxillary premolar or molar areas with a severe alveolar atrophy and a residual bone ridge height between 2 and 3 mm. The exclusion criteria were severe illness, head and neck radiation therapy, chemotherapy, uncontrolled diabetes or periodontal disease, smoking, sinus pathology, or presence of a residual root in the maxillary sinus. At the initial visit, all subjects underwent a clinical and occlusal examination, and panoramic radiographs were evaluated. A three-dimensional radiographic tomography scan (CBCT) (Vatech Ipax 3D PCH-6500, Fort Lee, NJ, USA) was performed before the procedure on the subjects to evaluate any clinically relevant and radiographically evident pathologies such as mucosal thickening, allergic or odontogenic sinusitis, mucus-retaining cysts, partial to complete sinus obliteration, oroantral communications, antroliths, mucoceles, and mucopyoceles (Figure 3, Figure 4, Figure 5 and Figure 6). In this way, CBCT was also able to analyze the patency of the ostium and the osteomeatal complex. After a thorough preliminary examination, patients were elected for bone regeneration procedure including sinus augmentation and implant insertion. Preoperatively, they were extensively informed concerning the surgical procedures.

**Figure 8:**
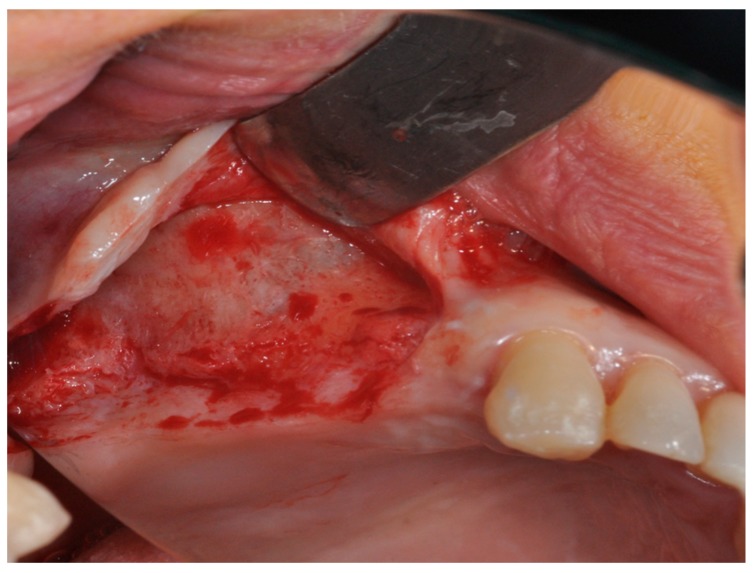
The modified triangular flap with a relieving incision in the distal region without a mesial relieving incision was performed.

**Figure 9:**
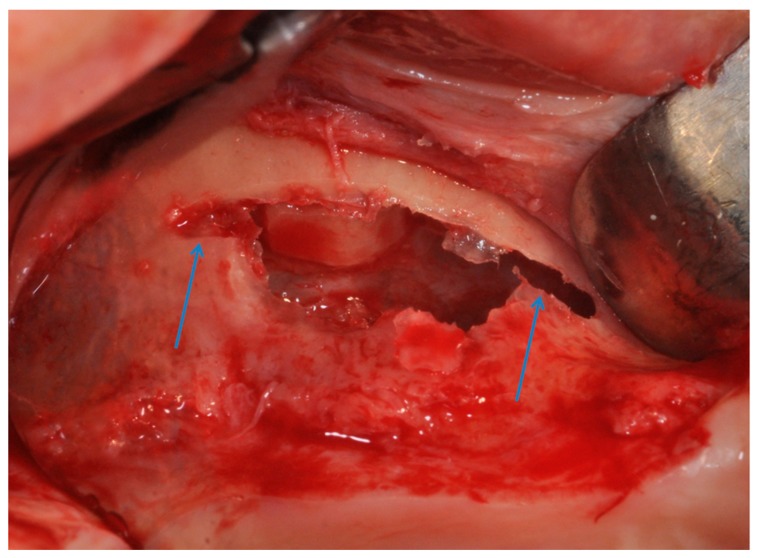
The antrostomy was performed with two lines of 2–3 mm, mesial and distal, was created at the top of the antrostom and sinus membrane was elevated.

Prior to surgery, the subjects’ mouths were rinsed with a chlorhexidine digluconate solution 0.2% for 2 min. Local anesthesia was performed by Articaine® (Ubistesin 4%-Espe Dental AG Seefeld, Germany) with epinephrine 1:100.000. A modified triangolar flap without anterior release, recently described by Scarano et al., was used [32]. In edentolous cases, the incision was performed on the top of the alveolar ridge horizontally and extended mesially, while, in other cases, the incision was intrasulcular starting near the mesialbuccal edge of the teeth and then extended up to the midpoint of the buccal sulcus of the canine, preserving the dental papilla (Figure 8) [32]. A full thickness flap was elevated to approach the lateral wall of the maxillary sinus and a trap door was made by piezoelectric unit device (Piezosurgery, Mectron, Carasco, Italy) under cold (4–5 °C) sterile saline irrigation to approach the lateral sinus wall with a mesio-distal measure of 6 mm and an apico-coronal height measure of 6 mm, starting from 2 mm from the crestal side; then, the bony door was removed (Figure 9). The elevation of the sinus membrane was accomplished by initially exposing and mobilizing the membrane using the ultrasonic handpiece followed by hand instrumentation to further elevate the membrane along all the walls of the sinus, and then it was elevated by dedicated curettes of different shapes, until it became completely detached from the lateral, inferior, and medial walls of the sinus.

In Group I, the sinuses were filled with collagened porcine bone (Geno-os, OsteoBiol, Turin, Italy); the graft was condensed at each stage and a collagen membrane (Evolution, OsteoBiol, Turin, Italy) was used to close the lateral window of the sinus.

In Group II, the heterologous barriers were washed with saline solution for 10 min.

**Figure 10:**
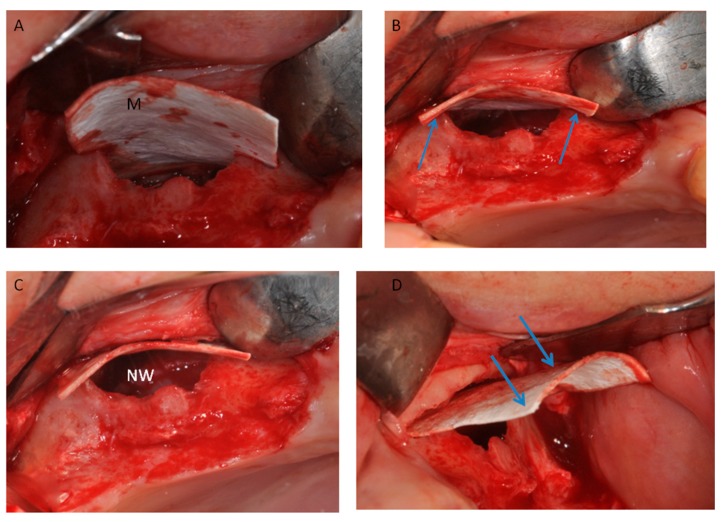
(**A**) Half of the heterologous membrane was positioned on on the top of wall. (**B**) The lamina was adapted on the two lines (Arrows); and (**C**) pushed to the nose wall of the sinus (NW). (**D**) Half of the heterologous membranes external of the sinus (arrows).

**Figure 11:**
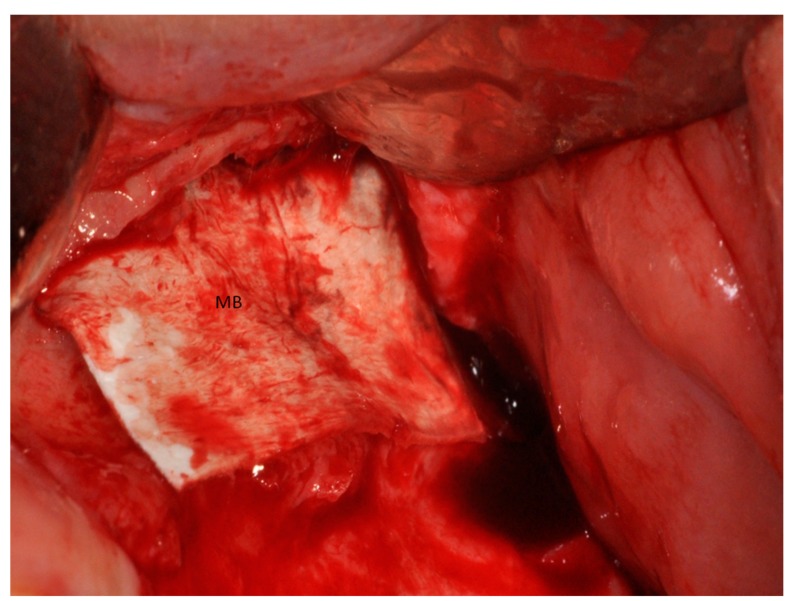
Half of the heterologous membranes external of the sinus was folded to cover the window (MB).

After contact, the bone lamina softens and it is possible to shape it allowing to fit the anatomic curvature of the sinus. The cortical laminar membrane was first softened and then imbibed with the patient’s blood for 2–3 min. In Group II, two lines of 2–3 mm, mesial and distal, were created at the top of the antrostomy (Figure 10). Half of the heterologous membrane was positioned on these lines and pushed to the nose wall of the sinus, and the other half was folded to cover the window (Figure 11). This procedure was performed carefully to avoid tearing or folding of the collagen membrane. The antrostomy was measured with a periodontal probe for cutting the membrane to size. Sinuses were randomly allotted into two groups, with 13 sinuses in each and a free random sampling and assignment application was used (Urbaniak, G.C., & Plous, S. 2013. Research Randomizer Version 4.0).

In Group II, the sinuses were treated with heterologous cortical lamina only (Lamina, OsteoBiol, Turin, Italy), and the coagulum was observed underneath the elevated sinus mucosa. The heterologous membrane was folded to cover the antrostomy. The operative time to complete the regenerative procedure (expressed in minutes) starting from antrostomy to the end of closing the lateral window was calculated.

CBCT scans were performed in pre-surgical phase and postoperative. DICOM dataset was analyzed by Ez3D Plus Software (EZ3D Plus, VATECH Global Fort Lee, NJ, USA) to elaborate in 3D model specimens and find the perfect position and alignment of sinuses and biomaterial scaffolds with the bone. The tomographies were conducted before the surgery, to diagnose bone; immediately after the surgery (T1); and six months after sinus grafting (T2) since this period was recommended by the manufacturer for implant insertion. The data reconstruction was performed with 1.0 mm in thickness and 0.2 mm interval parameter under 110 kVp and 8 mA with a low dosage protocol. After selection of the appropriate area using a specific tool and 3D reconstruction by an experienced radiologist, the software measured the volume graft. ITK-SNAP (Penn Image Computing and Science Laboratory, University of Pennsylvania, Philadelphia, PA, USA) software could realize by tomographic scanning images a contour segmentation of anatomical structures, organs and tissues was used in the present study.

Semi-automatic segmentation in ITK-SNAP uses a two-stage pipeline, providing manual tools for outlining and quality control. The graft volumes were estimated by geometry contour evaluation and refined fuzzy two-sided thresholds.

**Figure 12:**
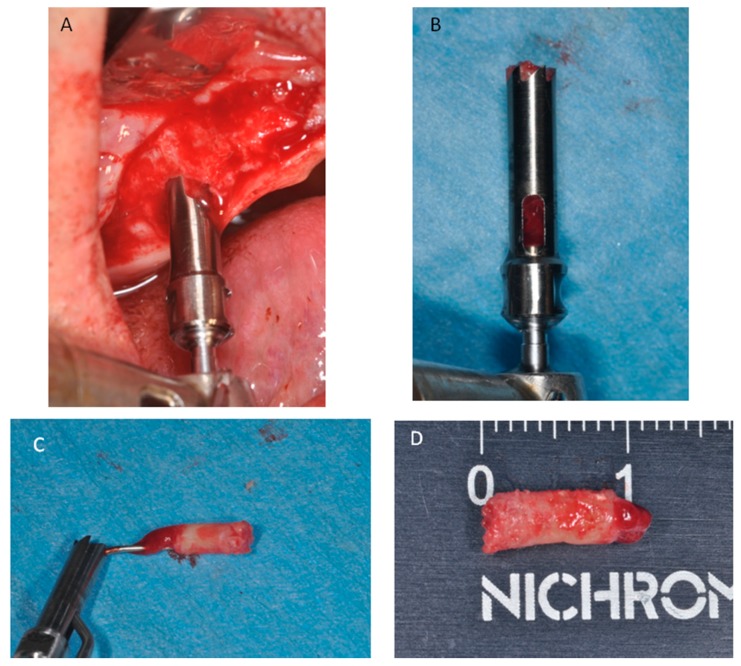
(**A**) After six months of healing a small trephine was used. (**B**) To retrieve a biopsy. (**C**) The bone core was removed from the trephine. (**D**) Bone core biopsy carried out.

Three-dimensional models of sinus grafted were generated and the imaging data were visualized in virtual space by MeshLab software (Visual Computing Lab-CNR ISTI, Pisa, Italy) to appreciate the changes of the graft volumes at different experimental times. The mean volume graft was 3101 ± 321 mm^3^ immediately postoperative and 2716.7 ± 432 mm^3^ after six months of healing (Figure 2). After a healing period of about six months, 34 implants (Bone System, Milano, Italy) were placed in the treated sinuses and the bone cores were harvested, before placement of the fixtures, using a 3.5 mm diameter trephine under cold (5–6 °C) sterile saline solution irrigation (Figure 12).

### Processing of Specimens

The bone cores were stored in 10% buffered formalin and processed for histology and histomorphometry at the Implant Retrieval Centre, Dental School, University of Chieti-Pescara, Italy in order to obtain thin ground sections with the Scan 1 Automated System (Pescara, Italy) [45]. Each sample was dehydrated in an ascending series of alcohol rinses and embedded in a glycolmethacrylate resin (Technovit 7200 VLC, Kulzer, Wehrheim, Germany). After completion of polymerization process, each specimen was sectioned longitudinally along its major axis with a high-precision diamond disc at about 150 µm and ground down to about 30 µm. Three slides were obtained for each specimen and stained with acid fuchsin and toluidine blue. The nomenclature approved by the American Society of Bone and Mineral Research was used to evaluate bone quality and histomorphometric measurements [46].

They were observed in normal transmitted light under a Nikon microscope ECLIPSE (Nikon, Tokyo, Japan). The different percentage of hard tissues, medullary space and biomaterials was carried out by a light microscope connected to a high resolution video camera (16.25-megapixel) (Digital Sight series microscope cameras), interfaced to a high definition monitor and a personal computer (Notebook Toshiba Satellite pro r50-c-15w). This optical system was associated with a histometry software package with image capturing capabilities, then recorded using a Sony α330 digital camera and subjected to morphometric analysis using digital image-analysis (NIS-Elements AR 3.0 software, Nikon, Minato, Japan).

## 5. Statistical Evaluation

A power analysis was accomplished using dedicated software freely available on the web (http://clincalc.com/stats/samplesize.aspx) to determine the numerosity of the sample needed to achieve statistical significance for quantitative analyses percentage of new bone formation and quantization of the percentage of the residual graft materials. A calculation model was adopted for dichotomous variables (yes/no effect) by putting the effect incidence designed to caution the reasons 25% for controls and 75% for treated, alpha was set at 0.05 and power at 80%. The optimal numerosity of sites for the evaluation was 14 sinuses for each experimental group. Differences between groups of treatment were determined by one-way analysis of variance (ANOVA) followed by Fisher’s Protected Least Significant Difference (PLSD) post-hoc test. Statistical evaluation was conducted using the Statview software from SAS Institute and a *p* value <0.05 was considered statistically significant.
